# Clinical features and factors associated with emergency department transfer among outpatients

**DOI:** 10.1097/MD.0000000000050467

**Published:** 2026-09-11

**Authors:** Fan Yang, Yiying Zhu, Qing Shao

**Affiliations:** a Outpatient Department, The Second Affiliated Hospital Zhejiang University School of Medicine, Hangzhou, Zhejiang, China; b ICU, The Second Affiliated Hospital Zhejiang University School of Medicine, Hangzhou, Zhejiang, China.

**Keywords:** associated factors, case–control study, emergency department transfer, logistic regression, outpatient clinic, physiological deterioration, vital signs

## Abstract

Unplanned emergency department (ED) transfer from outpatient clinics represents an important escalation point that may reflect early physiological deterioration and limited ambulatory capacity for monitoring and urgent interventions. We conducted a retrospective matched case-control study of consecutive adult outpatients treated at our institution between January 1, 2021 and December 31, 2024. Cases were outpatients requiring ED transfer during or shortly after the index outpatient encounter, confirmed by transfer documentation and same-day ED registration. Controls were outpatients discharged without ED transfer and were selected using 1:1 matching by outpatient department/specialty, calendar time of visit, sex, and age within 5 years. The final analytic cohort included 2184 cases and 2184 matched controls (4368 patients); 106 encounters were excluded based on prespecified criteria. Abnormal vital signs (35.1%) and cardiopulmonary symptoms (30.3%) were the most common primary indications for ED transfer. Compared with controls, cases exhibited higher temperature, heart rate, respiratory rate, and blood pressure and lower SpO2. In multivariable logistic regression, variables independently associated with ED transfer included shorter symptom duration, cardiopulmonary or infection-related presenting complaints, diabetes mellitus, chronic pulmonary disease, and acute physiological derangements, particularly higher temperature and respiratory rate, lower peripheral oxygen saturation (SpO2), elevated systolic blood pressure, and altered mental status. These findings indicate that ED transfer from outpatient settings was associated primarily with objective markers of acute instability and selected comorbidity profiles. Causal inference or direct use as a prospective decision rule should not be drawn from this retrospective case-control analysis.

## 1. Introduction

Unplanned emergency department (ED) transfer from outpatient clinics is a clinically relevant escalation point that may reflect acute physiological deterioration, limited ambulatory capacity for monitoring or urgent intervention, and variability in transfer thresholds across clinicians and services. Such transitions can expose patients to delays, fragmented handover, and duplicated testing, while also contributing to ED crowding and downstream hospitalization. As outpatient care increasingly involves older adults and patients with multimorbidity, reliable recognition of acute instability at the ambulatory encounter is essential. Basic physiological domains, including temperature, respiratory rate, oxygenation, blood pressure, heart rate, and mental status, are therefore central to outpatient safety assessment.^[1–3]^

Infectious syndromes remain a major cause of acute deterioration, and structured recognition of sepsis and septic shock continues to be emphasized. Recent studies highlight the importance of early physiological derangement recognition and the potential role of systematic detection pathways and digital tools, although implementation challenges remain.^[4,5]^

Nevertheless, evidence on outpatient-to-ED transfer remains limited. Prior research has mainly focused on ED triage, inpatient deterioration, or post-discharge ED use, rather than the ambulatory decision point at which escalation is initiated. Referral decisions may also vary according to provider-level factors such as clinical experience and reasoning.^[6]^ Although recent models have explored short-term hospitalization risk after outpatient visits,^[7]^ clinically interpretable evidence is still needed to clarify the indications and patient- and presentation-level factors associated with ED transfer from outpatient care.

However, there remains a need for clinically interpretable characterization of the indications prompting ED transfer from outpatient care and the independent patient- and presentation-level factors associated with such transfers, using rigorous comparison groups and standardized variable definitions. The present retrospective case-control analysis addresses this gap by evaluating outpatient encounters requiring ED transfer and matched outpatient controls, with emphasis on demographics, comorbidities, presenting complaint categories, vital signs, and the subset of available laboratory and imaging findings within routine clinical practice.

## 2. Methods

### 2.1. Study design

This study was approved by the Ethics Committee of The Second Affiliated Hospital Zhejiang University School of Medicine. This retrospective case-control study enrolled consecutive outpatient clinic attendees who received care at our institution between January 1, 2021 and December 31, 2024. Cases were defined as outpatients who experienced clinical deterioration during or immediately after the outpatient encounter and underwent ED transfer for urgent evaluation and/or treatment, whereas controls were outpatients managed entirely in the ambulatory setting without ED transfer during the same visit. Eligible participants were adults (≥18 years) with complete electronic medical records for the index outpatient visit, including demographic information, presenting complaints, vital signs, comorbidity profiles, key laboratory and/or imaging results when available, and documentation of disposition. Patients were excluded if they were initially registered as ED patients, were directly admitted to inpatient wards without ED transfer, underwent ED transfer for nonclinical reasons (e.g., administrative routing or patient preference without medical indication), had trauma as the primary reason for transfer, were pregnant, left against medical advice before disposition, were transferred to an external hospital from the outpatient clinic, or had substantial missing data in predefined core variables required for assessment of variables associated with ED transfer. The study was conducted in accordance with the Declaration of Helsinki. Written informed consent was obtained from all participants or their legally authorized representatives when required by institutional policy for retrospective research.

### 2.2. Study population and case definitions

Study population source. We retrospectively screened all consecutive outpatient clinic encounters at our institution during the study period using the outpatient registration database and linked electronic medical records. Eligible encounters were those with complete documentation for demographics, presenting symptoms, vital signs, and disposition. The index visit was defined as the outpatient encounter during which the study outcome ED transfer was adjudicated. For patients with multiple outpatient visits within the study window, only the first visit meeting eligibility criteria was included as the index visit to ensure independence of observations.

Case definition (outpatients requiring ED transfer). The case group comprised outpatients who, during the outpatient encounter or within a short time after the visit, exhibited clinical deterioration or an acute medical condition requiring urgent escalation of care and were transferred to the ED based on a clinician’s documented decision. ED transfer was confirmed by the presence of a formal transfer order in the outpatient record, a nursing transfer note, and a corresponding ED registration record temporally linked to the index outpatient visit. Transfer indications were determined from contemporaneous clinical documentation and were categorized into prespecified clinical scenarios, including abnormal vital signs (hypotension, hypertension with end-organ symptoms, tachycardia, bradycardia, tachypnea, hypoxemia, or high fever), cardiopulmonary symptoms (chest pain, palpitations with hemodynamic instability, shortness of breath, wheezing with respiratory compromise), neurological symptoms (acute altered mental status, syncope, focal neurological deficits, new-onset seizure), hemorrhagic events (hematemesis, melena, hemoptysis, gross hematuria, uncontrolled external bleeding), and suspected severe infection (systemic inflammatory response with suspected infection, suspected sepsis, or rapidly progressive local infection requiring intravenous therapy). When multiple indications were documented, the primary indication was defined as the reason most directly prompting ED transfer as stated by the treating clinician.

Control definition and selection strategy (outpatients without ED transfer). The control group included outpatients evaluated during the same study period who completed ambulatory assessment and management and were discharged from the outpatient clinic without ED transfer on the index visit date. Controls were selected using 1:1 matching to reduce confounding by temporal and service-related factors. Matching variables were defined a priori and included outpatient department/specialty, calendar time of visit (same day when feasible; otherwise within the same week), sex, and age within a prespecified caliper of 5 years. If multiple eligible controls were available for a given case after matching, the control with the closest visit date to the corresponding case was selected. Matching reduced but could not eliminate residual confounding from unmeasured provider-, clinic-, and access-related factors.

### 2.3. Outcome measure

The primary outcome was transfer from the outpatient clinic to the ED during the index visit, recorded as a binary variable (yes/no). An ED transfer event was considered present when the outpatient record documented a clinician-initiated decision for ED evaluation and the patient subsequently completed ED registration on the same date as the index outpatient encounter. Patients who completed outpatient management and were discharged without ED registration were classified as non-transfer.

### 2.4. Candidate predictors

Candidate variables were prespecified based on clinical plausibility and data availability in the electronic medical record, and were extracted from documentation corresponding to the index visit. Demographic and anthropometric variables included age (years) and sex (male/female). Visit-related characteristics included outpatient department/specialty at presentation and visit timing, categorized by weekday versus weekend and daytime versus nighttime according to the institutional clinic scheduling system. Presenting complaint was derived from the triage or clinician intake note. Symptom duration was extracted from the history of present illness and recorded in hours or days as documented; when symptom duration was reported in ranges, the value most consistently documented by the treating clinician was used.

Comorbidities were assessed using preexisting diagnoses documented in the medical history and/or diagnostic codes recorded before or on the index visit date. Comorbidity variables included hypertension, diabetes mellitus, coronary artery disease, chronic pulmonary disease, chronic kidney disease, and malignancy. Each condition was coded as present if it was listed in the problem list, prior discharge diagnoses, or outpatient diagnostic coding, and absent if no documentation or coding evidence was identified in the available records. Physiological parameters were obtained from the first set of vital signs recorded at the index outpatient encounter, including body temperature (°C), heart rate (beats/minutes), respiratory rate (breaths/minutes), systolic and diastolic blood pressure (mm Hg), and peripheral oxygen saturation (SpO_2_ ), %. Mental status was abstracted from nursing triage documentation and clinician examination notes and categorized as alert versus altered consciousness based on recorded findings. Laboratory and imaging variables were included when performed during the index outpatient encounter prior to disposition. Laboratory measures comprised complete blood count parameters, C-reactive protein, cardiac troponin, arterial or venous blood gas indices (when available), and D-dimer. Imaging variables were extracted from radiology reports generated during the same encounter, and were coded according to whether findings indicating an acute process were documented in the report impression.

### 2.5. Statistical analysis

All statistical analyses were performed using IBM SPSS Statistics, version 26.0 (IBM Corp., Armonk). Continuous variables were assessed for distributional characteristics and are presented as mean ± standard deviation when approximately normally distributed, or as median with interquartile range when skewed. Categorical variables are summarized as counts and percentages. Between-group comparisons (cases requiring ED transfer vs. matched controls) were conducted using the independent-samples *t* test for normally distributed continuous variables and the Mann–Whitney *U* test for non-normally distributed continuous variables. Categorical variables were compared using the chi-square test or Fisher’s exact test, as appropriate. Univariable associations with ED transfer were examined using univariable logistic regression, reporting odds ratios (ORs) with 95% confidence intervals (CIs). Variables showing statistical significance in univariable analyses were entered into a multivariable logistic regression model to identify variables independently associated with ED transfer, and adjusted ORs with 95% CIs were reported. Because laboratory and imaging tests were ordered selectively according to clinical judgment, these variables were interpreted as descriptive or subset findings and were not used to support causal inference; the possibility of confounding by indication was explicitly considered in interpreting these results. Owing to the retrospective case–control design, ORs quantify associations with documented ED transfer and should not be interpreted as incidence estimates, causal effects, or a validated prospective decision rule. All tests were two-sided, and *P* < .05 was considered statistically significant.

## 3. Results

### 3.1. Study cohort and flow

During the study period, all consecutive outpatient encounters were screened to identify eligible case patients, defined a priori as outpatients who required ED transfer during the index visit. After applying the prespecified inclusion and exclusion criteria, 2184 outpatients requiring ED transfer were included as the case group for the primary analysis. Subsequently, using the predefined 1:1 matching strategy, 2184 controls were selected from outpatients who completed ambulatory management and were discharged without ED transfer during the same study period, yielding a final matched cohort of 4368 patients (2184 matched pairs). A total of 106 encounters were excluded before cohort assembly and matching. The reasons for exclusion were as follows: initial ED registration (n = 24), direct admission to inpatient wards without ED transfer (n = 18), nonclinical ED transfer (n = 12), trauma as the primary reason for transfer (n = 9), pregnancy-related encounters (n = 8), abnormal disposition including refusal of transfer or leaving against medical advice before disposition (n = 14), transfer to an external hospital with unverifiable ED outcomes (n = 7), and substantial missing data in predefined core variables required for assessment of variables associated with ED transfer (n = 14).

### 3.2. Summary of indications for emergency department transfer

In the case cohort (n = 2184), abnormal vital signs were the most frequently documented primary indication for ED transfer (766 patients, 35.1%), followed by cardiopulmonary symptoms (662 patients, 30.3%). Neurological symptoms and suspected severe infection contributed similar proportions of transfers (301 patients, 13.8% and 297 patients, 13.6%, respectively). Hemorrhagic events were least common, accounting for 158 transfers (7.2%).

### 3.3. Baseline characteristics

In this matched cohort, cases and controls were similar in age and sex (both *P* > .05). The proportion of encounters with missing anthropometric data differed between groups. Visit timing characteristics (weekday/weekend and daytime/nighttime) and the distribution of outpatient departments did not differ materially. In contrast, the distribution of presenting complaint categories differed significantly, with a higher proportion of cardiopulmonary and infection-related presentations among cases. Symptom duration was shorter in cases than controls by nonparametric comparison. Regarding comorbidity profiles, cases had higher prevalences of hypertension, diabetes mellitus, coronary artery disease, chronic pulmonary disease, chronic kidney disease, and malignancy, and a higher overall comorbidity count, indicating a greater chronic disease burden among outpatients who required ED transfer (Table 1).

**Table 1:** Baseline demographic, visit-related, and comorbidity characteristics in cases and matched controls.

Variable	Cases (ED transfer) (n = 2184)	Controls (no ED transfer) (n = 2184)	Test statistic	*P* value
Age, yr	62.4 ± 13.1	62.5 ± 13.0	*t* = -0.15	.884
Male sex, n (%)	1121 (51.3)	1153 (52.8)	*χ*^2^ = 0.94	.332
Outpatient department, n (%)			*χ*^2^ = 5.64	.228
Internal medicine	971 (44.5)	984 (45.1)		
Surgery	416 (19.0)	432 (19.8)		
Cardiology	359 (16.4)	324 (14.8)		
Respiratory	223 (10.2)	221 (10.1)		
Other	215 (9.8)	223 (10.2)		
Weekend visit, n (%)	401 (18.4)	371 (17.0)	*χ*^2^ = 1.42	.234
Nighttime visit, n (%)	241 (11.0)	252 (11.5)	*χ*^2^ = 0.28	.599
Presenting complaint category, n (%)			*χ*^2^ = 132.45	<.001
Cardiopulmonary	723 (33.1)	431 (19.7)		
Gastrointestinal	309 (14.1)	383 (17.5)		
Neurological	232 (10.6)	205 (9.4)		
Infection-related	394 (18.0)	320 (14.7)		
Other	526 (24.1)	845 (38.7)		
Symptom duration, d	1.62 [0.86–2.96]	2.81 (1.51–5.18)	*U* = 1417	<.001
Symptom duration missing, n (%)	114 (5.2)	135 (6.2)	*χ*^2^ = 1.88	0.171
Hypertension, n (%)	1051 (48.1)	943 (43.2)	*χ*^2^ = 10.76	0.001
Diabetes mellitus, n (%)	521 (23.9)	389 (17.8)	*χ*^2^ = 24.19	<.001
Coronary artery disease, n (%)	350 (16.0)	272 (12.5)	*χ*^2^ = 11.41	<.001
Chronic pulmonary disease, n (%)	303 (13.9)	195 (8.9)	*χ*^2^ = 26.44	<.001
Chronic kidney disease, n (%)	177 (8.1)	134 (6.1)	*χ*^2^ = 6.40	.011
Malignancy, n (%)	131 (6.0)	90 (4.1)	*χ*^2^ = 8.01	.005
Comorbidity count	1.00 (0.00–2.00)	1.00 (0.00–1.00)	U = 2711	<.001

ED = emergency department, IQR = interquartile range.

### 3.4. Physiological and diagnostic findings

Physiological parameters differed between groups at the index outpatient encounter (Table 2). Cases had higher temperature, heart rate, respiratory rate, and blood pressure than controls (all *P* < .001), whereas SpO_2_ was lower among cases (*P* < .001). Consistently, abnormal vital sign thresholds were more frequent in cases, including high fever, tachycardia, tachypnea, and hypoxemia (all *P* < .001). Altered mental status was also more common among cases (5.2% vs 2.1%; χ^2^ = 30.00, *P* < .001), while hypotension was uncommon and did not differ significantly between groups (2.5% vs 1.9%; χ^2^ = 1.78, *P* = .182). Diagnostic testing was performed more often in cases than controls, including complete blood count, C-reactive protein, troponin, blood gas analysis, and D-dimer (all *P* < .001). Among tested patients, cases exhibited higher white blood cell counts and C-reactive protein concentrations, lower hemoglobin, and higher troponin and D-dimer levels (all *P* < .001). Biomarker elevations were more frequent in cases for both troponin and D-dimer (both *P* < .001), and blood gas results indicated greater physiological derangement among cases, with lower pH and higher lactate levels (both *P* < .001) (Table 2).

**Table 2:** Physiological parameters, testing completion, and laboratory results at the index outpatient visit.

Domain/Variable	Cases (ED transfer)	Controls (no ED transfer)	Test statistic	*P* value
Physiological parameters (continuous)				
Temperature, °C	37.4 ± 0.8 (n = 2184)	36.9 ± 0.5 (n = 2184)	*t* = 26.86	<.001
Heart rate, beats/min	95.4 ± 18.0 (n = 2184)	82.1 ± 14.7 (n = 2184)	*t* = 26.73	<.001
Respiratory rate, breaths/min	20.4 ± 4.0 (n = 2184)	17.3 ± 3.0 (n = 2184)	*t* = 28.67	<.001
Systolic blood pressure, mm Hg	138.8 ± 24.4 (n = 2184)	128.3 ± 18.1 (n = 2184)	*t* = 16.15	<.001
Diastolic blood pressure, mm Hg	82.3 ± 14.0 (n = 2184)	77.9 ± 12.1 (n = 2184)	*t* = 10.96	<.001
SpO_2_, %	93.9 ± 3.3 (n = 2184)	96.5 ± 1.9 (n = 2184)	*t* = -31.76	<.001
Physiological abnormalities (binary thresholds)				
High fever (≥38.0 °C), n (%)	496 (22.7)	24 (1.1)	*χ*^2^ = 486.33	<.001
Tachycardia (≥100 beats/min), n (%)	860 (39.4)	250 (11.4)	*χ*^2^ = 449.44	<.001
Tachypnea (≥22 breaths/min), n (%)	755 (34.6)	128 (5.9)	*χ*^2^ = 558.03	<.001
Hypotension (SBP < 90 mm Hg), n (%)	55 (2.5)	42 (1.9)	*χ*^2^ = 1.78	.182
Hypoxemia (SpO_2_ < 92%), n (%)	643 (29.4)	21 (1.0)	*χ*^2^ = 687.11	<.001
Altered mental status, n (%)	114 (5.2)	46 (2.1)	*χ*^2^ = 30.00	<.001
Testing completion at index visit				
Complete blood count performed, n (%)	1579 (72.3)	817 (37.4)	*χ*^2^ = 536.78	<.001
C-reactive protein performed, n (%)	1242 (56.9)	479 (21.9)	*χ*^2^ = 558.21	<.001
Cardiac troponin performed, n (%)	589 (27.0)	179 (8.2)	*χ*^2^ = 265.57	<.001
Blood gas analysis performed, n (%)	285 (13.0)	95 (4.3)	*χ*^2^ = 104.05	<.001
D-dimer performed, n (%)	719 (32.9)	254 (11.6)	*χ*^2^ = 285.91	<.001
Laboratory results among tested patients				
White blood cell count, ×10^9^/L	10.1 ± 3.8 (n = 1579)	7.7 ± 2.4 (n = 817)	*t* = 18.94	<.001
Hemoglobin, g/L	123.8 ± 18.3 (n = 1579)	130.5 ± 16.1 (n = 817)	*t* = −9.11	<.001
Platelet count, ×10^9^/L	237.3 ± 79.8 (n = 1579)	251.6 ± 72.4 (n = 817)	*t* = −4.43	<.001
C-reactive protein, mg/L	18.23 [10.56–31.98] (n = 1242)	6.23 [3.91–9.91] (n = 479)	*U* = 502,157	<.001
Cardiac troponin, ng/L	17.88 [9.23–32.10] (n = 589)	8.23 [4.92–13.58] (n = 179)	*U* = 78,063	<0.001
Troponin elevation (>34 ng/L), n/N (%)	139/589 (23.6)	6/179 (3.4)	*χ*^2^ = 36.75	<.001
pH	7.38 ± 0.06 (n = 285)	7.41 ± 0.04 (n = 95)	*t* = −6.62	<.001
Lactate, mmol/L	2.00 [1.44–3.00] (n = 285)	1.39 [1.02–1.73] (n = 95)	*U* = 19,394	<.001
D-dimer, mg/L FEU	1.14 [0.55–2.05] (n = 719)	0.45 [0.27–0.84] (n = 254)	*U* = 133,378	<.001
D-dimer elevation (>0.50 mg/L FEU), n/N (%)	554/719 (77.1)	115/254 (45.3)	*χ*^2^ = 88.22	<.001

ED = emergency department, FEU = fibrinogen equivalent units, IQR = interquartile range, SBP = systolic blood pressure, SpO_2_ = peripheral oxygen saturation, U = Mann–Whitney *U* statistic.

### 3.5. Univariable associations with ED transfer

Presenting complaint patterns differed, with cardiopulmonary and infection-related complaints showing higher odds of ED transfer compared with other complaints (OR 2.10 and 1.55, respectively; both *P* < .001), whereas gastrointestinal complaints were inversely associated (OR 0.70, *P* < .001). Several comorbidities were associated with ED transfer, including hypertension, diabetes mellitus, coronary artery disease, chronic pulmonary disease, chronic kidney disease, and malignancy (all *P* ≤ .001). Physiological derangements at presentation were strongly associated with ED transfer, including higher temperature, heart rate, and respiratory rate, lower SpO_2_, and altered mental status (all *P* < .001). Threshold-based abnormalities, high fever, tachycardia, tachypnea, and hypoxemia were also associated with substantially increased odds of ED transfer (all *P* < .001), whereas weekend and nighttime visits were not significantly associated. In tested subsets, higher C-reactive protein and elevations of troponin or D-dimer were associated with increased odds of ED transfer (all *P* < .001) (Table 3)

**Table 3:** Univariable logistic regression of factors associated with ED transfer.

Predictor (univariable)	OR	95% CI	Wald *z*	*P* value
Visit-related factors				
Weekend visit (yes vs no)	1.09	0.98–1.22	1.54	.125
Nighttime visit (yes vs no)	0.95	0.84–1.08	−0.75	.456
Symptom duration, per 1 d increase	0.80	0.77–0.83	−11.15	<.001
Presenting complaint category (reference: Other)				
Cardiopulmonary vs Other	2.10	1.87–2.36	12.37	<.001
Gastrointestinal vs Other	0.70	0.61–0.80	−5.10	<.001
Neurological vs Other	1.18	1.03–1.35	2.36	.018
Infection-related vs Other	1.55	1.38–1.74	7.30	<.001
Comorbidities				
Hypertension (yes vs no)	1.22	1.11–1.33	4.15	<.001
Diabetes mellitus (yes vs no)	1.40	1.26–1.56	6.09	<.001
Coronary artery disease (yes vs no)	1.30	1.16–1.47	4.36	<.001
Chronic pulmonary disease (yes vs no)	1.63	1.41–1.87	6.75	<.001
Chronic kidney disease (yes vs no)	1.32	1.11–1.56	3.18	.001
Malignancy (yes vs no)	1.48	1.19–1.83	3.54	<.001
Physiological parameters at presentation				
Temperature, per 1 °C increase	2.77	2.42–3.18	14.57	<.001
Heart rate, per 10 beats/min increase	1.50	1.41–1.59	13.50	<.001
Respiratory rate, per 1 breath/min increase	1.20	1.18–1.23	15.42	<.001
SpO_2_, per 1% increase	0.70	0.68–0.72	−19.83	<.001
Altered mental status (yes vs no)	2.55	1.92–3.39	6.47	<.001
Vital-sign thresholds (binary)				
High fever ≥ 38.0°C (yes vs no)	21.12	13.85–32.18	14.19	<.001
Tachycardia ≥ 100 beats/min (yes vs no)	3.74	3.23–4.34	17.60	<.001
Tachypnea ≥ 22 breaths/min (yes vs no)	8.00	6.52–9.83	19.81	<.001
Hypoxemia SpO_2_ < 92% (yes vs no)	29.37	18.71–46.10	14.70	<.001
Laboratory predictors (tested subsets)				
CRP, per 10 mg/L increase (tested subset)	1.30	1.23–1.38	8.73	<.001
Troponin elevation > 34 ng/L (tested subset)	7.17	3.15–16.33	4.69	<.001
D-dimer elevation > 0.50 mg/L FEU (tested subset)	3.63	2.60–5.07	7.59	<.001

CRP = C-reactive protein, ED = emergency department, FEU = fibrinogen equivalent units, OR = odds ratio, SpO_2_ = peripheral oxygen saturation.

### 3.6. Variables independently associated with ED transfer

In the multivariable model restricted to variables showing statistical significance in univariable analyses (Table 4), longer symptom duration was independently associated with lower odds of ED transfer (adjusted OR 0.86 per 1-day increase, 95% CI 0.83–0.89, *P* < .001). Compared with other presenting complaints, cardiopulmonary presentations (adjusted OR 1.62, 95% CI 1.41–1.87, *P* < .001) and infection-related presentations (adjusted OR 1.28, 95% CI 1.11–1.47, *P* = .001) were associated with higher odds of ED transfer, whereas gastrointestinal complaints were associated with lower odds (adjusted OR 0.82, 95% CI 0.70–0.96, *P* = .015). Diabetes mellitus (adjusted OR 1.18, 95% CI 1.05–1.34, *P* = .006) and chronic pulmonary disease (adjusted OR 1.31, 95% CI 1.12–1.53, *P* < .001) remained significant comorbidity-related variables. Among physiological parameters, higher temperature and respiratory rate, lower SpO_2_, higher systolic blood pressure, and altered mental status were independently associated with ED transfer (all *P* ≤ .012).

**Table 4:** Multivariable logistic regression for independent risk factors associated with ED transfer.

Predictor (retained)	Adjusted OR	95% CI	Wald *z*	*P* value
Symptom duration, per 1-d increase	0.86	0.83–0.89	−8.52	<.001
Presenting complaint category (reference: Other)				
Cardiopulmonary vs Other	1.62	1.41–1.87	6.20	<.001
Infection-related vs Other	1.28	1.11–1.47	3.30	.001
Gastrointestinal vs Other	0.82	0.70–0.96	−2.43	.015
Comorbidities				
Diabetes mellitus (yes vs no)	1.18	1.05–1.34	2.74	.006
Chronic pulmonary disease (yes vs no)	1.31	1.12–1.53	3.43	<.001
Physiological parameters at presentation				
Temperature, per 1 °C increase	1.90	1.63–2.21	7.31	<.001
Respiratory rate, per 1 breath/min increase	1.09	1.07–1.12	8.63	<0.001
SpO_2_, per 1% increase	0.79	0.77–0.82	−15.88	<.001
Systolic blood pressure, per 10 mm Hg increase	1.05	1.01–1.09	2.52	.012
Altered mental status (yes vs no)	1.74	1.22–2.49	3.05	.002

CI = confidence interval, ED = emergency department, OR = odds ratio, SpO_2_ = peripheral oxygen saturation.

## 4. Discussion

This retrospective case–control study evaluated clinical features and independent risk factors associated with ED transfer among outpatients, using a large, 1:1 matched cohort. The main findings were that outpatient-to-ED escalation was most frequently triggered by abnormal vital signs and cardiopulmonary presentations, and that multivariable-adjusted associations were dominated by markers of acute physiological instability at the index visit, including higher body temperature, higher respiratory rate, lower SpO2, elevated systolic blood pressure, and altered mental status. In addition, shorter symptom duration, cardiopulmonary and infection-related presenting complaints, diabetes mellitus, and chronic pulmonary disease remained independently associated with ED transfer. Collectively, these results support a risk profile in which acute deterioration signals, rather than demographic characteristics alone, are central to identifying outpatients requiring urgent escalation.

A principal contribution of this work is the quantitative characterization of “transfer triggers” in ambulatory care. The predominance of abnormal vital signs and cardiopulmonary symptoms among ED transfers is clinically coherent, as these presentations often reflect imminent threats to oxygenation, ventilation, perfusion, or cardiac stability. Respiratory rate and oxygen saturation, in particular, were strongly associated with ED transfer in adjusted analyses. This aligns with contemporary evidence emphasizing the prognostic value of basic physiological measures and early warning score components for short-term clinical deterioration and need for urgent intervention. In emergency care populations, NEWS2 has been evaluated as a tool for predicting time-critical treatment requirements, underscoring that physiological derangements can serve as actionable signals for escalation pathways.^[8,9]^ Although our study focused on outpatient encounters rather than ED triage, the directionality is consistent: respiratory compromise and global instability are recurrent determinants of high-acuity trajectories.

Fever and infection-related complaints also emerged as independent correlates of ED transfer, suggesting that suspected systemic infection remains a major driver of escalation from outpatient settings. This is relevant in contemporary ambulatory practice, where clinicians frequently face diagnostic uncertainty and must balance watchful waiting against timely escalation. The independent association between higher temperature and ED transfer may reflect both the clinical severity of infection syndromes and the operational reality that outpatient clinics often have limited capacity for prolonged monitoring, intravenous therapies, or rapid diagnostic escalation. In this context, structured assessment tools and standardized transfer processes may reduce variation in decision-making and improve safety. A recent quality improvement initiative targeting outpatient-to-ED transfers highlighted the importance of early recognition and standardized stabilization and handover procedures, supporting the broader concept that process design is a modifiable determinant of transfer safety.^[10,11]^

The observed associations of diabetes mellitus and chronic pulmonary disease with ED transfer may reflect reduced physiological reserve and greater susceptibility to decompensation during acute illness. Patients with diabetes may present with infection-related complications, metabolic decompensation, and a higher risk of adverse outcomes when physiological stress is present. Chronic pulmonary disease plausibly increases the probability that relatively modest acute insults respiratory infection, bronchospasm, or fluid overload translate into hypoxemia or tachypnea, which in turn prompts escalation. Importantly, while several comorbidities were associated with ED transfer in univariable analyses, only a subset remained significant in the adjusted model. This pattern supports the interpretation that comorbidities act partly through acute physiological expression at presentation. From a practical standpoint, comorbidity information remains valuable for contextualizing risk, but the results suggest that immediate physiological assessment is essential for risk stratification in real time.^[12]^ Another notable finding was the inverse association between symptom duration and ED transfer, with shorter duration associated with higher odds of escalation. This may indicate that abrupt symptom onset is interpreted by clinicians as more concerning for acute cardiopulmonary or neurological events, leading to ED referral. Alternatively, shorter symptom duration may act as a proxy for higher acuity symptom evolution, which cannot be safely managed within routine outpatient workflows.^[13]^ These interpretations are consistent with outpatient clinical reasoning, where rapid progression and unclear diagnosis frequently trigger ED transfer to expedite diagnostic imaging, laboratory testing, and continuous monitoring.

The results have implications for ambulatory safety systems. First, the independent predictive value of respiratory rate, oxygen saturation, temperature, and mental status supports the use of structured physiological screening at outpatient triage or intake, especially in high-volume clinics. Second, because early warning tools show variable performance across settings and implementations, the selection and governance of scoring systems require careful evaluation. A large comparative study of early warning scores spanning both simple aggregated scores and proprietary models demonstrated substantial heterogeneity in accuracy and highlighted the need for transparency and oversight.^[14]^ Although these studies were conducted in inpatient contexts, the central inference is applicable: prediction tools should be validated for the target setting and linked to clear response pathways. In addition, recent syntheses have emphasized that while many studies validate NEWS/NEWS2 prognostic accuracy, fewer evaluate whether implementation improves patient outcomes, indicating that effectiveness depends on coupling risk detection with operational response and clinical action.^[15]^ For outpatient settings, this implies that physiological screening is necessary but not sufficient; the escalation pathway, handover quality, and receiving ED readiness remain critical to achieving benefit. Third, our findings support prioritizing standardized transfer and handoff practices for outpatient-to-ED transitions. Improvements in handoff quality have been associated with better care quality indicators in prehospital-to-ED transitions, suggesting that structured communication strategies can yield measurable process improvements.^[16]^ Although outpatient-to-ED workflows differ from ambulance handoffs, both involve inter-team communication under time pressure, and both are vulnerable to information loss. Implementation strategies may include standardized transfer documentation, explicit recording of transfer indication category, and structured vital sign summaries. The quality improvement literature on outpatient transfers further supports system-level interventions that incorporate staff training and standardized processes.

This study has limitations that should be considered when interpreting the findings. First, the retrospective design is subject to misclassification and documentation bias, particularly for variables derived from clinical notes, such as symptom duration and the primary indication for ED transfer when multiple triggers co-occur. Second, laboratory and imaging data were incompletely available and likely influenced by selective testing; consequently, associations involving biomarkers may reflect both disease severity and clinician testing behavior, representing potential confounding by indication. Third, although 1:1 matching was used to reduce confounding by age, sex, clinic specialty, and visit timing, residual confounding remains possible. Important unmeasured factors may include clinician experience and risk tolerance, outpatient clinic crowding, local availability of monitoring or urgent diagnostic resources, socioeconomic status, and access to follow-up care. Fourth, as a single-institution study, external validity may be limited because outpatient workflows, triage systems, and thresholds for ED transfer differ across health systems. Future work should prospectively evaluate the operational performance of physiological screening and escalation pathways in outpatient clinics, including calibration across clinic types and patient subgroups. Pragmatic studies comparing structured early warning-informed triage with usual care, combined with standardized ED transfer and handoff bundles, would help clarify whether these strategies reduce adverse events, delays, or unnecessary transfers. In addition, future model development should prioritize interpretability and implementation feasibility, given that tool effectiveness depends on clinician uptake and well-defined response actions.

## 5. Conclusion

In conclusion, ED transfer among outpatients was primarily associated with acute physiological instability and high-acuity symptom complexes, particularly cardiopulmonary and infection-related presentations. These findings support the integration of structured physiological assessment with standardized escalation and handoff procedures in outpatient settings to improve the timeliness and safety of ED transfer decisions.

## Author contributions

**Conceptualization:** Fan Yang, Yiying Zhu, Qing Shao.

**Data curation:** Fan Yang, Yiying Zhu, Qing Shao.

**Formal analysis:** Fan Yang, Yiying Zhu, Qing Shao.

**Funding acquisition:** Fan Yang.

**Investigation:** Fan Yang.

**Writing – original draft:** Fan Yang.

**Writing – review & editing:** Fan Yang.
